# Radioembolization with Intra-Arterial Angiotensin II to Improve Tumor-Absorbed Dose (RADIANT): A Single-Arm Phase II Study Protocol

**DOI:** 10.3390/cancers18193129

**Published:** 2026-09-27

**Authors:** Niek Wijnen, Marnix G. E. H. Lam, Khalil Ramdhani, Rutger C. G. Bruijnen, Martijn M. A. Dietze, Camiel E. M. Kerckhaert, Hugo W. A. M. de Jong, Maarten L. J. Smits

**Affiliations:** Department of Radiology and Nuclear Medicine, University Medical Center Utrecht, 3584 CX Utrecht, The Netherlands

**Keywords:** angiotensin II, colorectal cancer liver metastases, hepatocellular carcinoma, liver tumors, radioembolization, study protocol, tumor-to-non-tumor ratio, yttrium-90

## Abstract

Purpose: The effectiveness of transarterial radioembolization (TARE) depends largely on the tumor-to-non-tumor ratio (TNR): the ratio of microsphere concentration between tumor and non-tumorous liver tissue. Angiotensin II (AT-II) may improve TNR by inducing vasoconstriction primarily in arteries supplying healthy liver parenchyma. Tumor-feeding arteries, lacking neurovascular innervation, are largely unresponsive to AT-II, potentially resulting in preferential blood flow redistribution toward tumor tissue. The RADIANT study aims to evaluate whether intra-arterial AT-II infusion prior to yttrium-90 (^90^Y) microsphere injection improves tumor selectivity during TARE in patients with primary or metastatic liver tumors. Materials and Methods: This single-center, single-arm phase II study will include 15 patients with primary or metastatic liver tumors (any histological subtype, diameter ≥ 2 cm) referred for ^90^Y glass radioembolization. Each patient serves as their own control: during work-up, technetium-99m macroaggregated albumin (^99m^Tc-MAA) is administered without AT-II and TNR is measured on SPECT/CT. During the therapeutic procedure, AT-II is infused intra-arterially (10 µg/min over 100 s) immediately before ^90^Y microsphere injection, and TNR is measured on post-treatment ^90^Y PET/CT. The primary endpoint is the TNR improvement factor (TNR after ^90^Y + AT-II divided by TNR after ^99m^Tc-MAA). Secondary endpoints include toxicity and technical success of AT-II administration. Discussion: The RADIANT study will provide prospective evidence on the effect of intra-arterial AT-II infusion on tumor selectivity during ^90^Y radioembolization.

## Simple Summary

Radioembolization is an established treatment for primary and metastatic liver tumors in which radioactive microspheres are administered through the hepatic arteries. Treatment efficacy depends on selective microsphere deposition within tumor tissue while limiting radiation exposure to healthy liver parenchyma. Angiotensin II causes stronger vasoconstriction in healthy hepatic arteries than in tumor-feeding vessels and may therefore temporarily redirect arterial blood flow and microspheres toward tumors. The prospective RADIANT phase II study will investigate whether intra-arterial angiotensin II infusion improves tumor selectivity during yttrium-90 radioembolization. Fifteen patients will undergo a standard technetium-99m macroaggregated albumin work-up without angiotensin II, followed by yttrium-90 treatment with angiotensin II infusion. Tumor-to-normal liver absorbed-dose ratios will be compared between the two procedures, and technical success and safety will also be assessed. If angiotensin II safely improves tumor-selective microsphere delivery, this strategy may increase the tumor absorbed dose and provide a basis for larger disease-specific studies evaluating clinical outcomes.

## 1. Introduction

Transarterial radioembolization (TARE) is an established minimally invasive locoregional treatment for primary or metastatic liver tumors [1,2]. Its effectiveness largely depends on the tumor-to-non-tumor ratio (TNR), defined as the ratio of microsphere concentration in tumor tissue to that in non-tumorous liver parenchyma. TNRs typically range from 1 to 4, but substantial interindividual heterogeneity exists, and microsphere biodistribution may vary between tumors even within the same patient [3,4,5]. This variability can reduce treatment effectiveness and increase toxicity to healthy liver parenchyma. Low TNRs are especially worrisome in patients who undergo whole-liver or lobar treatment or in patients with poor liver function. Therefore, there is a need to increase tumor selectivity and elevate the TNR in TARE.

Angiotensin II (AT-II) may offer a means to improve TNR. AT-II is an endocrine vasoconstrictor that activates smooth muscle cells in the arterial vessel wall [6]. Liver tumor vasculature is often immature, and tumor-feeding arteries typically lack neurovascular innervation and have poorly developed smooth muscle cells [7,8]. As a result, tumor-feeding arteries show limited responsiveness to AT-II compared with arteries supplying healthy liver parenchyma. This differential responsiveness provides an opportunity to manipulate hepatic arterial hemodynamics: following local administration of AT-II, vasoconstriction occurs primarily in arteries supplying non-tumorous liver parenchyma, thereby redirecting blood flow toward tumor tissue [9,10]. In theory, this could increase TNR and improve the efficacy of radioembolization.

This approach has previously been investigated in five human studies, all of which reported a significant increase in TNR following hepatic arterial AT-II administration, with median TNR improvement factors (i.e., TNR after AT-II infusion divided by TNR prior to AT-II infusion) ranging from 1.8 to 3.1 [4,9,10,11,12,13]. These findings suggest that AT-II can enhance microsphere distribution toward tumor tissue. However, these studies are outdated and were conducted using imaging methods and radioactive tracers that are not applied in current clinical practice, limiting their applicability to modern radioembolization.

Current radioembolization practice relies on hybrid imaging (SPECT/CT and yttrium-90 [^90^Y] PET/CT) and advanced dosimetry software to quantify TNR with greater accuracy. Therefore, the objective of this study is to assess the potential vasomodulatory benefit of hepatic arterial AT-II infusion within a contemporary clinical TARE workflow.

## 2. Materials and Methods

### 2.1. Study Design

The RADIANT study is designed as a prospective, investigator-initiated, single-center, single-arm phase II clinical trial conducted at University Medical Center Utrecht (Utrecht, The Netherlands). Each patient serves as their own control by undergoing a work-up procedure without AT-II and a therapeutic procedure with AT-II infusion, enabling within-patient TNR comparison. The trial started patient recruitment in January 2026. The trial was registered in the European Union Clinical Trials Information System (CTIS; EU Trial Number 2025-521870-33-00) and at ClinicalTrials.gov (NCT07312292).

### 2.2. Study Population

Patients aged ≥18 years with confirmed primary or metastatic liver tumors of any histological subtype, with at least one tumor measuring ≥2 cm in diameter, and a referral for ^90^Y radioembolization as part of standard care, are eligible. The full inclusion and exclusion criteria are provided in Table 1.

### 2.3. Investigational Medicinal Product

The investigational medicinal product is angiotensin II, commercially available as Giapreza (PAION Deutschland GmbH, Aachen, Germany) as a 2.5 mg/mL concentrate for solution for infusion [14]. Giapreza holds EMA marketing authorization for intravenous treatment of refractory hypotension in adults with septic or distributive shock; its use in the RADIANT study as an intra-arterial hepatic infusion constitutes an off-label application. The mechanism by which AT-II selectively increases hepatic tumor blood flow is illustrated in Figure 1.

In this study, AT-II is administered at 10 µg/min over 100 s (total dose ~17 µg per injection position) via the same microcatheter and at the exact same injection position used for subsequent ^90^Y microsphere injection. This local intra-arterial delivery ensures that AT-II acts directly on the hepatic vasculature supplying the target territory, consistent with all previous human studies evaluating AT-II-mediated improvement of microsphere distribution [4]. The dose and duration were selected based on a prior dose-escalation study, demonstrating that 10 µg/min produced a more pronounced TNR improvement than 5 µg/min, and that TNR peaks at approximately 100 s after initiation of infusion [10]. Given AT-II’s plasma half-life of less than one minute, ^90^Y glass microspheres are injected immediately upon completion of AT-II infusion to ensure microsphere distribution occurs during peak AT-II-mediated tumor hyperemia.

### 2.4. Interventions

Included patients will undergo a standardized radioembolization treatment protocol that consists of a work-up procedure and a therapeutic procedure. AT-II will only be infused during the therapeutic procedure, prior to microsphere injection. A flowchart of the study procedures is represented in Figure 2.

### 2.5. Work-Up Procedure

The work-up procedure is performed according to standard clinical protocol. Under local anesthesia, the femoral or radial artery is punctured, an arterial sheath is placed, and the hepatic arteries are catheterized. Hepatic vascular anatomy is mapped with digital subtraction angiography and CBCT is acquired at each desired injection position to determine the perfusion territory. A total scout dose of approximately 150 MBq technetium-99m macroaggregated albumin (^99m^Tc-MAA) is then administered without AT-II infusion, divided over the planned injection positions when multiple positions are used. Same-day SPECT/CT will assess ^99m^Tc-MAA distribution, lung shunting, and extrahepatic deposition. If intrahepatic distribution is considered inadequate for treatment, or if clinically relevant extrahepatic deposition cannot be corrected, the patient will be excluded from treatment and from the study.

### 2.6. Therapeutic Procedure

The therapeutic procedure will be performed no more than 3 weeks after the ^99m^Tc-MAA work-up. During the therapeutic procedure, ^90^Y glass microspheres (TheraSphere; Boston Scientific Marlborough, MA, USA) will be administered from the same injection position(s) as the work-up. After the correct catheter position and the vascular anatomy have been reconfirmed by digital subtraction angiography, AT-II will be infused intra-arterially at 10 µg/min for 100 s and immediately followed by ^90^Y microsphere injection. If the work-up injection position cannot be reproduced, AT-II will not be administered to prevent catheter position mismatch from confounding the TNR comparison; the patient will instead receive ^90^Y microsphere injection according to standard clinical protocol and will be excluded from the primary analysis. If multiple injection positions are used, AT-II will be administered at 10 µg/min for 100 s at each position. If whole-liver treatment is split into two sessions to reduce liver toxicity, AT-II will be administered before ^90^Y injection in both sessions. Post-treatment ^90^Y PET/CT, performed one day after the procedure, will be used to quantify microsphere distribution.

### 2.7. Imaging Protocols

Pre-treatment ^99m^Tc-MAA planar and SPECT/CT imaging will be performed on a dual-headed SPECT/CT camera (Symbia Pro.specta X3; Siemens Healthineers, Erlangen, Germany). Planar imaging will be acquired to assess lung shunting and extrahepatic deposition. SPECT will be acquired using a low-energy high-resolution collimator, a 256 × 256 matrix, 120 projections over a 360° non-circular orbit, 20 s per projection, and a 140 keV ± 7.5% energy window. Images will be reconstructed using 3D ordered-subset expectation maximization (3D OSEM; 48 iterations, 1 subset) with CT-based attenuation correction, scatter correction, and a 10 mm Gaussian post-reconstruction filter. The accompanying low-dose CT will be acquired at 110 kVp with 1.5 mm slice thickness and used for attenuation correction and anatomical co-registration.

Post-treatment ^90^Y PET/CT will be performed one day after radioembolization on a PET/CT system (Biograph Trinion.X EP9 CT128; Siemens Healthineers, Erlangen, Germany). PET images will be acquired over the treated liver according to the institutional ^90^Y PET/CT protocol and reconstructed using time-of-flight, point-spread-function, ordinary Poisson ordered-subset expectation maximization (TOF-PSF-OP-OSEM; 4 iterations, 6 subsets) with a 4 mm Gaussian post-reconstruction filter. Images will be corrected for normalization, attenuation, scatter, random coincidences, dead time, and radioactive decay. The accompanying low-dose CT (90–140 kVp, 3 mm slice thickness) will be used for attenuation correction and anatomical co-registration.

A contrast-enhanced CT (ceCT) of the liver will be acquired alongside both the pre-treatment ^99m^Tc-MAA SPECT/CT and the post-treatment ^90^Y PET/CT. The ceCT will be performed in the arterial phase, the portal venous phase, or both. The arterial phase will be acquired with a 20 s delay after administration of 125 mL intravenous contrast at 4 mL/s, and the portal venous phase with a 49 s delay after administration of 125 mL intravenous contrast at 3 mL/s.

### 2.8. Safety Monitoring and Adverse-Event Follow-Up

Patients are monitored with continuous heart rate, oxygen saturation, and respiratory rate throughout the therapeutic procedure and for one hour afterwards. Blood pressure is measured before the procedure, every 5 min during the procedure, and at one hour post-procedure. In the event of excessively elevated blood pressure or intolerable symptoms, AT-II infusion is immediately discontinued. Due to AT-II’s half-life of less than one minute, symptoms are expected to resolve rapidly.

Despite the short plasma half-life of AT-II (<1 min), study-specific safety surveillance will continue until 7 days after the therapeutic procedure. During this period, the patient’s electronic health record will be reviewed to identify any healthcare contacts, reported symptoms, or clinical findings. All adverse events, serious adverse events, and suspected unexpected serious adverse reactions occurring during this period will be recorded and graded according to CTCAE version 5.0. For each event, the investigator will assess seriousness, expectedness, and its potential causal relationship to AT-II administration. No additional study-specific clinical visit or laboratory assessment is scheduled; patients receive the same follow-up as standard-of-care radioembolization, which includes a telephone consultation at 1–2 weeks post-procedure to assess for new or ongoing symptoms. Additional clinical assessment or laboratory testing will be performed when clinically indicated. All adverse events will be followed until resolution or until a stable clinical situation has been reached.

### 2.9. Study Objectives

The primary objective of the RADIANT study is to evaluate whether intra-arterial AT-II infusion increases tumor selectivity during ^90^Y TARE in patients with primary or metastatic liver tumors. This is assessed by comparing TNR after ^99m^Tc-MAA injection without AT-II versus TNR after ^90^Y glass microsphere injection with AT-II. The primary endpoint is the patient-level TNR improvement factor:

TNR improvement factor=TNR90Y + AT−II (measured on Y90 PET/CT)TNR99mTc−MAA (measured on SPECT/CT)

To calculate the TNRs, three categories of volumes of interest (VOIs) will first be delineated within each hepatic perfusion territory: (1) the perfused liver parenchyma within the perfusion territory, (2) tumor VOI(s), and (3) the healthy liver tissue (HLT) VOI, defined as the perfused liver parenchyma within the perfusion territory minus the tumor VOI. To account for potential interval tumor growth between the work-up and therapeutic procedures, VOIs will be segmented on both the ceCT acquired alongside the post-scout ^99m^Tc-MAA SPECT/CT and the ceCT acquired alongside the post-treatment ^90^Y PET/CT, and subsequently co-registered to the low-dose CT of the corresponding SPECT/CT or PET/CT using Simplicit90Y software (Mirada Medical, Oxford, UK).

Following segmentation and co-registration of the VOIs, VOI-based multicompartment dosimetry will also be performed using Simplicit90Y, with the mean estimated absorbed dose (Gy) quantified separately in the segmented tumor and healthy liver tissue VOIs on both pre-treatment ^99m^Tc-MAA SPECT/CT and post-treatment ^90^Y PET/CT. TNR will be calculated as the mean estimated absorbed dose in the tumor VOI(s) divided by the mean estimated absorbed dose in the corresponding HLT VOI, derived from the respective imaging modality for each time point. For patients with multiple tumor VOIs, perfusion territories, and/or treatment sessions, tumor and HLT absorbed-dose measurements are pooled separately using volume weighting to obtain a single patient-level TNR at each imaging time point, as detailed in the Statistical methods section. The TNR calculation workflow is illustrated in Figure 3.

Secondary endpoints are the toxicity profile of intra-arterial AT-II infusion, graded according to the Common Terminology Criteria for Adverse Events (CTCAE) version 5.0 [15], and technical success, defined as successful intra-arterial AT-II administration at 10 µg/min for 100 s immediately followed by ^90^Y microsphere injection.

### 2.10. Sample Size

A sample size calculation was performed to ensure adequate power to detect a clinically meaningful effect. Based on the prior studies demonstrating TNR improvement factors of 1.8–3.1 following AT-II infusion, a 50% multiplicative increase in TNR (i.e., TNR after ^90^Y + AT-II = 1.5 × TNR after ^99m^Tc-MAA) was considered the minimum clinically relevant effect [4]. The primary analysis will use log-transformed TNR values; the target effect corresponds to a mean difference of log(1.5) ≈ 0.41 on the log scale. The standard deviation of the paired log-transformed TNR values was estimated to be 0.52, based on a previous radioembolization trial conducted at our center in 21 patients with CRLM [16]. This dataset was selected because it represented the closest available data from our institution that used a similar paired within-patient framework, a comparable VOI-based image-analysis and dosimetry approach with separate quantification of tumor and healthy liver tissue, and the same underlying tumor-to-non-tumor ratio concept. Using these parameters, with a two-sided alpha of 0.05 and 80% power, a paired t-test requires 15 patients.

Accordingly, the target sample size refers to 15 patients with evaluable TNR measurements at both imaging assessments (i.e., TNR_99mTc-MAA_ and TNR_90Y+AT-II_), allowing calculation of the TNR improvement factor. If a participant does not provide an evaluable TNR pair because of withdrawal, technical failure, non-evaluable imaging, incomplete treatment, or another cause of missing TNR data, the missing measurement will not be imputed and an additional participant will be enrolled. Recruitment will continue until 15 patients with evaluable TNR pairs have been included to preserve the prespecified statistical power. All enrolled participants and reasons for non-evaluability will be reported.

### 2.11. Statistical Methods

Participant demographics and baseline disease characteristics will be summarized using descriptive statistics. Categorical variables will be reported as counts and percentages. Continuous variables will be reported as mean ± standard deviation if normally distributed, or as median with interquartile range if non-normally distributed.

The primary endpoint is the patient-level TNR improvement factor. Because the treatment effect is expressed as a relative (multiplicative) change in TNR, TNR values from both time points (after ^99m^Tc-MAA injection without AT-II infusion and after ^90^Y radioembolization with AT-II infusion) will be log-transformed prior to analysis.

The patient will be the primary unit of analysis. In patients with multiple tumor VOIs within a perfusion territory, a volume-weighted mean tumor absorbed dose will be calculated across all tumor VOIs. For patients with multiple perfusion territories and/or treatment sessions, the tumor VOIs and corresponding healthy liver tissue VOIs will be pooled across all treated territories and sessions, and volume-weighted mean absorbed doses will be calculated separately for tumor and healthy liver tissue. The patient-level TNR will subsequently be calculated as the volume-weighted mean tumor absorbed dose divided by the volume-weighted mean healthy liver tissue absorbed dose. This approach will provide one patient-level TNR at each imaging time point (i.e., TNR_99mTc-MAA_ and TNR_90Y+AT-II_) and, consequently, one paired TNR improvement factor per patient. In addition, a lesion-based analysis will be performed using the index lesion, defined as the tumor with the largest diameter.

Given the within-patient design, the paired log-difference will be calculated as log(TNR_90Y+AT-II_) − log(TNR_99mTc-MAA_). Normality of the paired log-differences will be assessed using the Shapiro–Wilk test, with *p* < 0.05 considered evidence against normality. If normality is supported, the mean paired log-difference and its 95% confidence interval (CI) will be estimated using a paired t-test. If normality is not supported, a percentile bootstrap procedure with 10,000 patient-level resamples will be used to obtain the 95% CI for the observed mean paired log-difference, with complete patient pairs resampled to preserve the paired structure. In both cases, the observed mean paired log-difference and the lower and upper limits of its 95% CI will be exponentiated and reported as the geometric mean TNR improvement factor with its corresponding 95% CI, representing the average fold-change in TNR with AT-II infusion. The TNR improvement will be considered statistically significant if the 95% CI of the geometric mean TNR improvement factor excludes 1.

The primary analysis will include all patients irrespective of tumor type. Given the limited sample size, the study is not powered to detect differences in the effect of AT-II between tumor types. Two prespecified exploratory subgroup analyses are planned: one according to tumor origin (primary versus metastatic) and one according to histological diagnosis (hepatocellular carcinoma [HCC], colorectal cancer liver metastases [CRLM], or other). Subgroup-specific geometric mean TNR improvement factors will be summarized descriptively, with 95% CIs (calculated as described above) reported where estimable. No formal hypothesis testing between subgroups will be performed.

Technical success and safety of AT-II infusion, the secondary endpoints, will be summarized as counts and percentages. The technical success rate and the incidence of each adverse event (graded according to CTCAE v5.0) will be reported with two-sided exact 95% binomial CIs calculated using the Clopper–Pearson method.

## 3. Discussion

The RADIANT study is a prospective phase II trial designed to evaluate whether intra-arterial AT-II infusion at 10 µg/min for 100 s improves tumor selectivity during ^90^Y radioembolization for primary or metastatic liver tumors, with the TNR improvement factor as the primary endpoint.

There is robust evidence that a higher tumor-absorbed dose is strongly associated with improved disease control and overall survival (OS) in patients treated with TARE [17,18,19,20]. In HCC, the prospective multicenter PROACTIF cohort of 989 patients treated with ^90^Y glass microspheres demonstrated a strong dose–survival relationship: median OS was 16.8 months in patients with a pretreatment absorbed dose to the index lesion < 200 Gy versus 26.0 months in those receiving ≥200 Gy, and 19.7 months in patients receiving <400 Gy versus 30.7 months in those receiving ≥400 Gy [17]. A similar dose–response relationship has been demonstrated for CRLM: a study of 31 patients treated with glass ^90^Y microspheres showed that median tumor-absorbed dose was higher in lesions with complete (196 Gy) or partial (177 Gy) metabolic response than in lesions with progressive disease (95 Gy) [20]. These data underscore that tumor-absorbed dose is a key determinant of TARE effectiveness and that strategies capable of increasing the TNR by delivering higher doses to the tumor have the potential to directly improve patient outcomes.

Given the association between tumor-absorbed dose and clinical outcome, strategies to increase tumor-selective microsphere deposition have been investigated. One approach is using modified catheters for activity infusion. For instance, balloon catheters (e.g., Occlusafe^®^, Terumo Europe NV, Leuven, Belgium; or Sniper^®^, Embolx, Sunnyvale, CA, USA) or catheters with an umbrella-shaped tip (Surefire^®^ or Trinav^®^, Trisalus Life Sciences, Westminster, CO, USA). The hypothesis for these catheters is that they improve tumor targeting through reduced downstream pressure, potentially redirecting flow toward tumor tissue. However, prospective comparative evidence remains limited and has not demonstrated improved tumor targeting. Moreover, in a within-patient randomized study of 21 CRLM patients treated with holmium-166 TARE, the use of the Surefire^®^ anti-reflux catheter did not result in a higher TNR compared with a standard microcatheter (median TNR 3.2 vs. 3.6, respectively), nor did it improve tumor-absorbed dose or metabolic response [16]. More recently, a prospective within-patient study of seven patients undergoing ^166^Ho TARE found that balloon occlusion resulted in a lower TNR in six patients (geometric mean ratio, 0.41; *p* = 0.06) and was associated with incomplete tumor coverage [21]. The need for strategies that reliably increase tumor selectivity therefore remains unmet.

AT-II infusion offers a mechanistically distinct approach: rather than modifying catheter fluid dynamics, it exploits the differential vascular responsiveness between normal and tumor-feeding hepatic arteries to redirect blood flow toward the tumor. This mechanism is supported by consistent preclinical and clinical evidence demonstrating significant TNR improvements following hepatic arterial AT-II administration [9,10,11,12,13,22,23]. Nonetheless, hepatic arterial AT-II infusion has not been further evaluated in contemporary radioembolization practice after these early studies. This lack of follow-up was likely related to the withdrawal of AT-II formulations from the European and United States markets, leaving no approved AT-II product available for clinical use for many years. With the introduction of Giapreza, a synthetic human AT-II formulation, clinical re-evaluation of intra-arterial AT-II infusion in TARE has become feasible. The RADIANT study is therefore the first to evaluate this approach using imaging and dosimetry methods applied in current radioembolization practice.

AT-II infusion is anticipated to carry a low risk of serious adverse events. In the five prior human studies, the only consistently reported adverse effect was a transient increase in systolic blood pressure, up to 40 mmHg in some patients, sometimes accompanied by hypertension-related symptoms such as headache or a feeling of chest oppression [4]. In all patients, blood pressure returned to baseline and symptoms resolved within minutes after AT-II infusion was ceased, and no adverse event was severe enough to necessitate premature discontinuation. To ensure patient safety, vital parameters are monitored continuously throughout the procedure. If excessively elevated blood pressure or intolerable symptoms occur, AT-II infusion is immediately discontinued.

The main limitation of this study is that TNR comparisons are made across two different imaging modalities (SPECT/CT and PET/CT) and two different tracers (^99m^Tc-MAA and ^90^Y glass microspheres). ^99m^Tc-MAA preparations consist of heat-denatured albumin aggregates with heterogeneous particle-size distributions (typical range: 10–90 μm) and complex, nonspherical morphology, whereas TheraSphere consists of spherical glass microspheres within a diameter range of 20–30 μm, with ^90^Y incorporated into the glass matrix [24,25]. These differences in particle composition, size, and morphology may affect intrahepatic transport and microvascular deposition independently of AT-II. These particle-related differences, together with differences in spatial resolution between SPECT/CT and PET/CT and changes in hepatic flow dynamics between the work-up and therapeutic procedures, contribute to the known imperfect correspondence between ^99m^Tc-MAA and ^90^Y microsphere distributions [26,27,28]. Consequently, an observed TNR improvement cannot be attributed exclusively to AT-II. Although published studies have demonstrated heterogeneous findings regarding agreement between pretreatment ^99m^Tc-MAA SPECT/CT and post-treatment ^90^Y PET/CT dosimetry, some studies using ^90^Y glass microspheres have reported comparable mean dosimetric measurements [29,30,31,32]. For example, Ho et al. reported mean TNRs of 5.6 ± 3.2 on pretreatment ^99m^Tc-MAA SPECT/CT and 5.9 ± 3.5 on post-treatment ^90^Y PET/CT in 62 HCC patients [29]. Similarly, Kafrouni et al. reported comparable mean tumor doses (169 ± 40 versus 165 ± 47 Gy) and healthy-liver doses (36 ± 16 versus 34 ± 15 Gy) on ^99m^Tc-MAA SPECT/CT and ^90^Y PET/CT, with concordance correlation coefficients of 0.86 and 0.90, respectively [30]. Across these studies, differences between the reported group means for the evaluated dosimetric parameters were approximately 2–6%. Taken together, these findings do not indicate consistent systematic overestimation or underestimation of dosimetric measurements derived from ^99m^Tc-MAA SPECT/CT relative to ^90^Y PET/CT when averaged across patients. A geometric mean TNR improvement of 50% would therefore be unlikely to result solely from systematic tracer- or modality-related bias. However, this does not imply that an improvement factor of 1.5 lies outside the full methodological variability, as substantial individual-level variability and other between-session factors may still contribute. Accordingly, the findings will be interpreted as exploratory rather than as definitive evidence of an AT-II effect [31].

Another limitation is the small sample size of 15 patients. However, this limitation is partly mitigated by the within-patient design, in which each patient undergoes both the work-up procedure without AT-II and the therapeutic procedure with AT-II, thereby serving as their own control. By accounting for interindividual variability in patient demographics, tumor characteristics, and hepatic arterial anatomy, the paired design reduces the sample size required to detect a meaningful effect and limits the influence of patient-level confounders on the primary analysis. Nevertheless, the standard deviation used for the sample size calculation was derived from a ^166^Ho radioembolization study conducted at our center in patients with CRLM. This dataset was selected to minimize variability arising from center-specific practices. However, its applicability to a mixed-tumor population treated with ^90^Y remains uncertain. RADIANT should therefore be regarded as an exploratory phase II study that may provide preliminary evidence to support the design of a subsequent, adequately powered trial.

Finally, the inclusion of both primary and metastatic liver tumors introduces additional heterogeneity. Although the within-patient design reduces the influence of between-patient variability on the primary comparison, the limited sample size precludes reliable conclusions regarding tumor-type-specific effects. The broad inclusion criteria are intentional and permit preliminary exploration of the effect of AT-II across different tumor types. Any observed differences between tumor-type subgroups will therefore be considered exploratory and hypothesis-generating and may inform the design of larger, disease-specific trials.

If a significant TNR improvement factor is demonstrated alongside an acceptable safety profile, the study would provide the contemporary evidence base needed to justify a larger, disease-specific trial. Such a trial would be required to determine whether AT-II should be routinely used to enhance the efficacy of modern radioembolization. If no meaningful TNR improvement is observed, this will be an equally important finding, suggesting that routine use of AT-II infusion is not justified.

## 4. Conclusions

In conclusion, RADIANT is an exploratory phase II trial that aims to provide contemporary evidence on whether intra-arterial AT-II infusion safely improves tumor selectivity during ^90^Y radioembolization in patients with primary or metastatic liver tumors. Any positive findings will require confirmation in a larger, disease-specific controlled trial before AT-II infusion could be considered for routine clinical use.

## Figures and Tables

**Figure 1:**
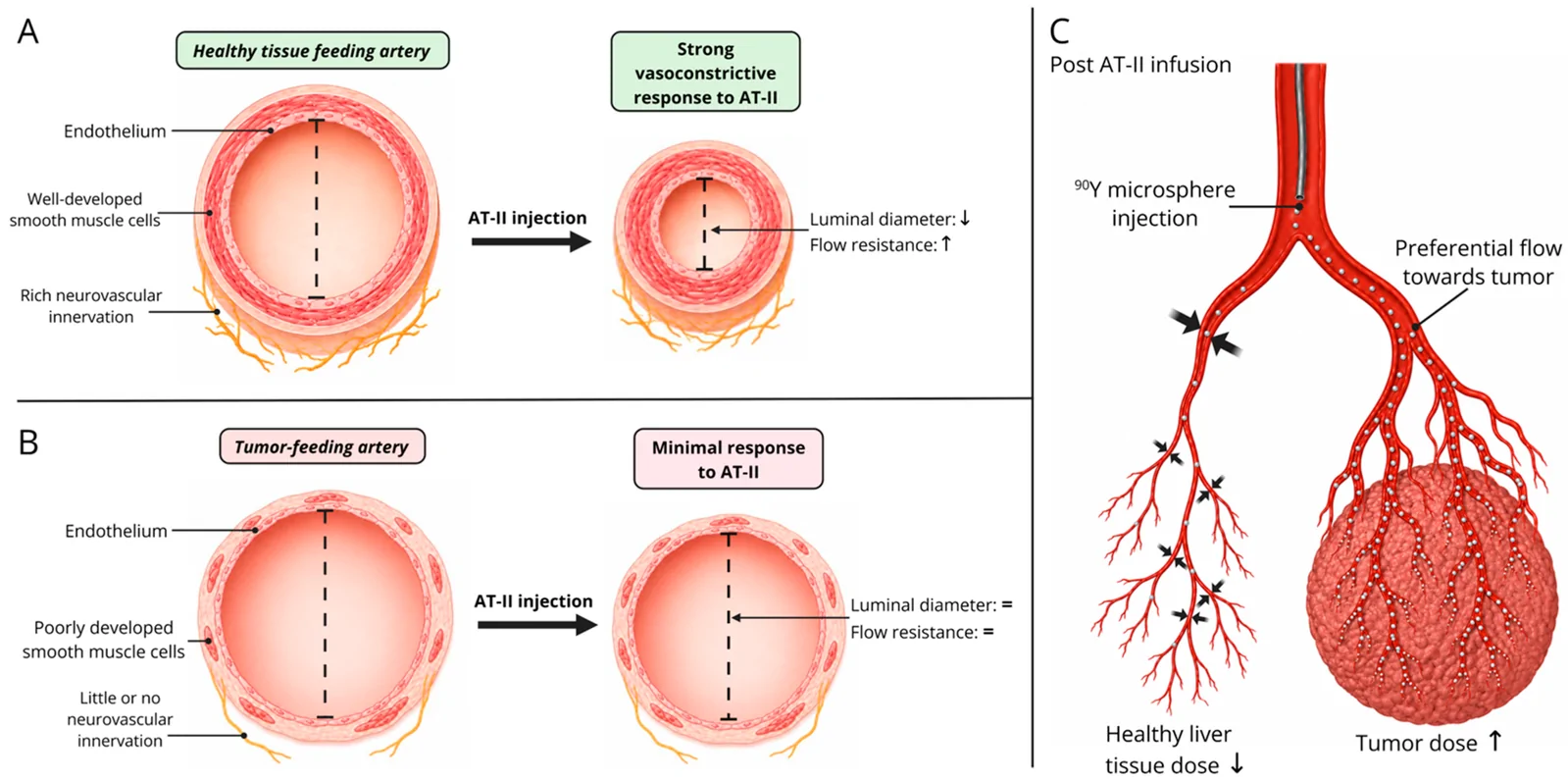
Mechanism of action of intra-arterial angiotensin II infusion in ^90^Y radioembolization. (**A**) Cross-section of a healthy tissue-feeding artery. Well-developed smooth muscle cells and rich neurovascular innervation render the vessel highly responsive to angiotensin II (AT-II). Following AT-II injection, smooth muscle contraction reduces luminal diameter and increases flow resistance, resulting in decreased arterial blood flow to healthy liver parenchyma. (**B**) Cross-section of a tumor-feeding artery. Poorly developed smooth muscle cells and little or no neurovascular innervation render the vessel largely unresponsive to AT-II. Following AT-II injection, luminal diameter and flow resistance remain unchanged, preserving arterial blood flow to the tumor. (**C**) Schematic simulation of hepatic arterial flow after intra-arterial AT-II infusion. Selective vasoconstriction of healthy liver-feeding arteries (indicated by black arrows) reduces parenchymal perfusion, while tumor-feeding arteries remain patent. This differential vascular response redirects arterial blood flow preferentially toward the tumor, resulting in increased tumor-absorbed dose and decreased healthy liver tissue dose following ^90^Y microsphere injection.

**Figure 2:**
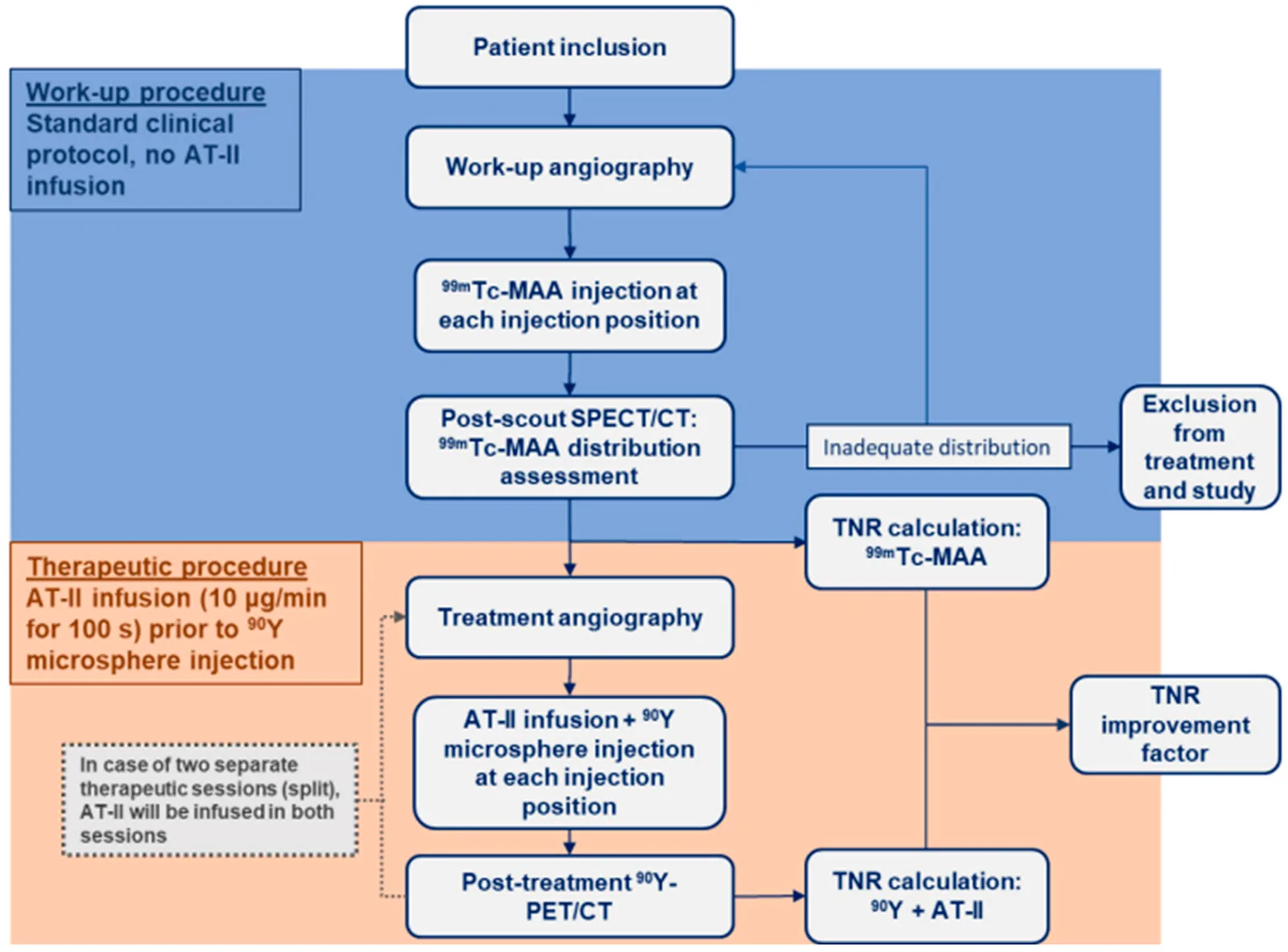
Flowchart illustrating the study design. Blue and orange squares represent the steps of the work-up and therapeutic procedure, respectively. During the work-up procedure, AT-II will not be infused. During the therapeutic procedure, at each injection position, AT-II will be infused (10 μg/min during 100 s), directly followed by injection of ^90^Y glass microspheres. In cases of two separate therapeutic procedures (split session), AT-II will be infused during both procedures (at each injection position).

**Figure 3:**
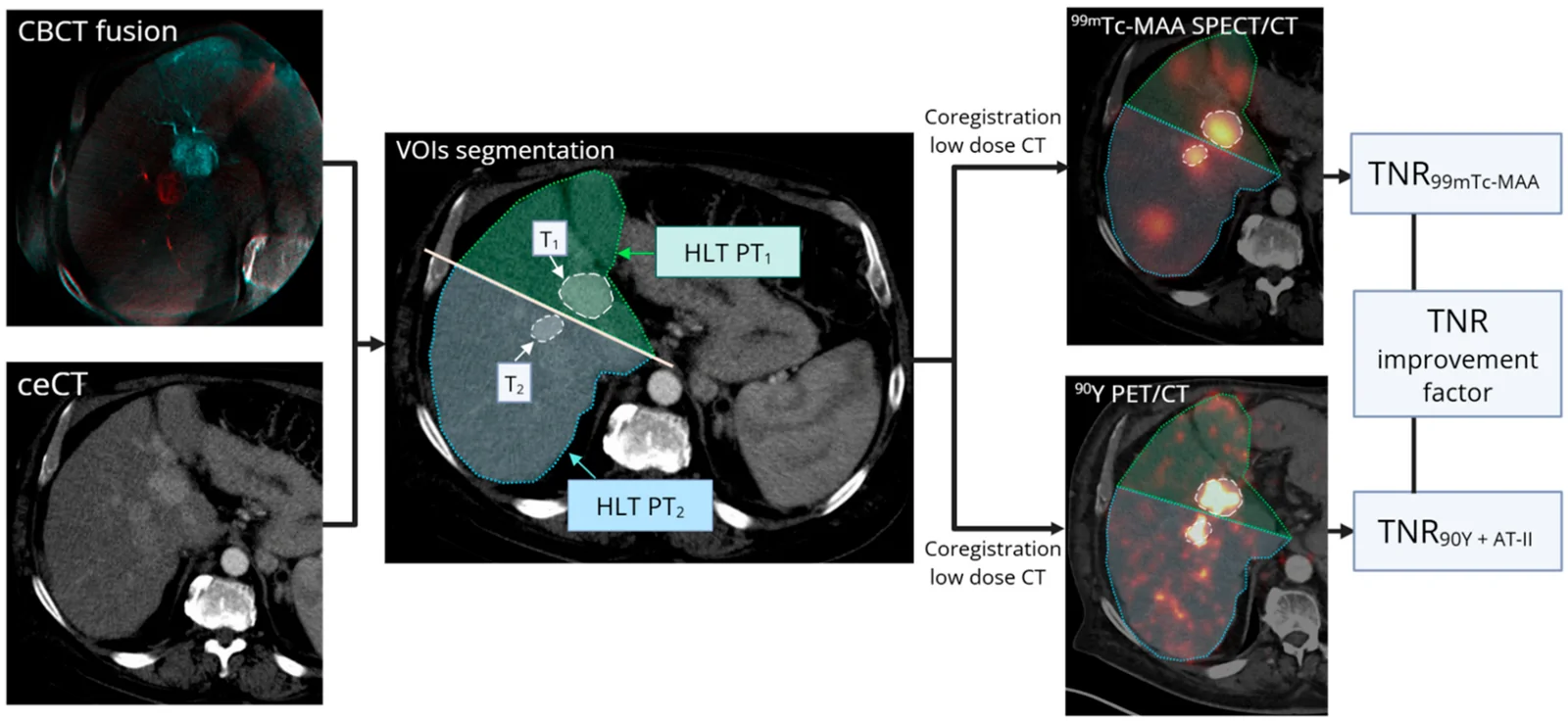
TNR calculation workflow in the RADIANT study. Example from a patient (not treated in this study) with bilobar multifocal HCC requiring two injection positions. First, perfusion territories (PT) are estimated for each injection position based on cone-beam CT (CBCT) acquired during intra-arterial contrast injection (PT_1_: blue overlay; PT_2_: red overlay). Volumes of interest (VOIs) are segmented semi-automatically on both the ceCT acquired alongside the post-scout ^99m^Tc-MAA SPECT/CT and the ceCT acquired alongside the post-treatment ^90^Y PET/CT: whole perfused liver parenchyma, tumor tissue (T), and healthy liver tissue (HLT) (i.e., perfused liver parenchyma VOI minus tumor VOI). The ceCT with VOIs is subsequently co-registered to the low-dose CT of the corresponding post-scout ^99m^Tc-MAA SPECT/CT or post-treatment ^90^Y PET/CT. At each imaging time point, estimated absorbed doses are quantified in the tumor and corresponding HLT VOIs within each perfusion territory. For patients with multiple tumor VOIs, perfusion territories, and/or treatment sessions, tumor and HLT absorbed-dose measurements are pooled separately using volume weighting. One patient-level TNR is then calculated at each imaging time point as the volume-weighted mean tumor absorbed dose divided by the volume-weighted mean HLT absorbed dose. The patient-level TNR improvement factor is calculated as the TNR with AT-II derived from post-treatment ^90^Y PET/CT (TNR_90Y+AT-II_) divided by the TNR without AT-II derived from post-scout 99mTc-MAA SPECT/CT (TNR_99mTc-MAA_).

**Table 1:** Eligibility criteria for RADIANT.

Inclusion Criteria	Exclusion Criteria
Primary or metastatic liver tumors (any histological type)	Serious comorbidity precluding safe AT-II administration, including: (1) uncontrolled hypertension; (2) treatment with ≥3 antihypertensive drugs; (3) arterial (cerebro-)vascular or venous thromboembolic event within the past 6 months
Clinical indication for radioembolization	Current use of ACE inhibitors or ARBs
Liver tumor diameter ≥ 2 cm	Known hypercoagulable state
Age ≥ 18 years	History of severe peripheral vascular disease
Competent and able to provide informed consent	Known hypersensitivity to AT-II or any Giapreza excipient *
Written informed consent	Serious and/or chronic liver disease precluding safe radioembolization
	Uncorrectable extrahepatic scout-dose deposition †
	Pregnancy or breastfeeding
	Body weight > 150 kg (table limit)
	Known severe allergy to contrast agents
	Participation in another investigational study that may compromise study endpoints
	Any other significant comorbidity or medical condition compromising radioembolization safety

* Giapreza excipients: mannitol, sodium hydroxide, and hydrochloric acid. † Activity in the falciform ligament, hepatoduodenal lymph nodes, and gallbladder is accepted. Abbreviations: ACE, angiotensin-converting enzyme; ARB, angiotensin II receptor blocker; AT-II, angiotensin II.

## Data Availability

The original contributions presented in this study are included in the article. Further inquiries can be directed to the corresponding author.
